# Supplementary material for: Distinct SoxB1 networks are required for naïve and primed pluripotency
Source: eLife. 2017 Dec 19;6:e27746. doi: 10.7554/eLife.27746 (PMC5758114; doi:10.7554/eLife.27746)
Supplement: Supplementary file 2. [file elife-27746-supp2.docx]

**Supplementary file 1: List of the cell lines used in this study.**

| **Line name** | ***Sox2* genotype** | ***Sox3*^¶^ genotype** | **Other transgenes** | **Parental line** |
| --- | --- | --- | --- | --- |
| E14Tg2a | *Sox2^+/+^* | *Sox3^WT^* |  | E14 (Hooper *et al*, 1987) |
| SCKO | *Sox2^fl/-^* | *Sox3^WT^* | CAG-CreER^T2^-ires-puroR* | CJ7 (Favaro *et al*, 2009) |
| S#R^§^ | *Sox2^-/-^* | *Sox3^WT^* | CAG-Sox#-ires-hygroR* | SCKO |
| SKO^§^ | *Sox2^-/-^* | *Sox3^WT^* | CAG-CreER^T2^-ires-puroR* | SCKO |
| S3KO^§^ | *Sox2^+/+^* | *Sox3^null^* |  | E14Tg2a |
| Clones 36, 37 | *Sox2^fl/-^* | *Sox3^null^* | CAG-CreER^T2^-ires-puroR* | SCKO |

^¶^on chromosome X, only one allele.

^§^population of cells, unless followed by clone number.

*randomly integrated transgene.
