# Supplementary material for: Distinct SoxB1 networks are required for naïve and primed pluripotency
Source: eLife. 2017 Dec 19;6:e27746. doi: 10.7554/eLife.27746 (PMC5758114; doi:10.7554/eLife.27746)
Supplement: Transparent reporting form [file elife-27746-transrepform.docx]

***eLife’s* transparent reporting form**

We encourage authors to provide detailed information *within their submission* to facilitate the interpretation and replication of experiments. If you have any questions, please contact us: [editorial@elifesciences.org](mailto:editorial@elifesciences.org).

**Sample-size estimation**

- You should state whether an appropriate sample size was computed when the study was being designed
- You should state the statistical method of sample size computation and any required assumptions
- If no explicit power analysis was used, you should describe how you decided what sample (replicate) size (number) to use

Please outline where this information can be found within the submission (e.g., page numbers or figure legends), or explain why this information doesn’t apply to your submission:

No experiments requiring sample-size estimation (such as *in vivo* experiments) were performed. Microarray data were generated using biological triplicates as normally accepted for such technique.

**Replicates**

- You should report how often each experiment was performed
- You should include a definition of biological versus technical replication
- The data obtained should be provided and sufficient information should be provided to indicate the number of independent biological and/or technical replicates
- If you encountered any outliers, you should describe how these were handled
- Criteria for exclusion/inclusion of data should be clearly stated
- High-throughput sequence data should be uploaded before submission, with a private link for reviewers provided (these are available from both GEO and ArrayExpress)

Please outline where this information can be found within the submission (e.g., page numbers or figure legends), or explain why this information doesn’t apply to your submission:

Information about number of biological replicates can be found in the figure legends throughout the manuscript. For microarray, detailed explanation of replicate numbers and statistical analysis can be found in the methods section.

Raw and analysed microarray data have been deposited at GEO under accession code GSE00195 (<https://www.ncbi.nlm.nih.gov/geo/query/acc.cgi?acc=GSE99185>; reviewer access token: qtmjkeoevvepxgr)

**Statistical reporting**

- Statistical analysis methods should be described and justified
- Raw data should be presented in figures whenever informative to do so (typically when N per group is less than 10)
- For each experiment, you should identify the statistical tests used, exact values of N, definitions of center, methods of multiple test correction, and dispersion and precision measures (e.g., mean, median, SD, SEM, confidence intervals; and, for the major substantive results, a measure of effect size (e.g., Pearson's r, Cohen's d)
- Report exact p-values wherever possible alongside the summary statistics and 95% confidence intervals. These should be reported for all key questions and not only when the p-value is less than 0.05.

For evaluation of variability in quantitative PCR experiments and alkaline phosphatase experiments, error bars were calculated as SEM, which considers the number of replicates for each experiment.

For TIDE analysis, we based our approach to identify significant indels using the parameters provided by the online TIDE tool and described in Brinkman et al, 2014.

Statistical analysis for microarray data is described in the methods section. Raw and analysed microarray data have been deposited at GEO under accession code GSE00195 (<https://www.ncbi.nlm.nih.gov/geo/query/acc.cgi?acc=GSE99185>; reviewer access token: qtmjkeoevvepxgr)

Please outline where this information can be found within the submission (e.g., page numbers or figure legends), or explain why this information doesn’t apply to your submission:

(For large datasets, or papers with a very large number of statistical tests, you may upload a single table file with tests, Ns, etc., with reference to page numbers in the manuscript.)

**Additional data files (“source data”)**

- We encourage you to upload relevant additional data files, such as numerical data that are represented as a graph in a figure, or as a summary table
- Where provided, these should be in the most useful format, and they can be uploaded as “Source data” files linked to a main figure or table
- Include model definition files including the full list of parameters used
- Include code used for data analysis (e.g., R, MatLab)
- Avoid stating that data files are “available upon request”

Please indicate the figures or tables for which source data files have been provided:

Analysed microarray data are available as Supplementary File 1.

Raw and analysed microarray data have been deposited at GEO under accession code GSE00195 (<https://www.ncbi.nlm.nih.gov/geo/query/acc.cgi?acc=GSE99185>; reviewer access token: qtmjkeoevvepxgr)
